# Expression of Tau40 Induces Activation of Cultured Rat Microglial Cells

**DOI:** 10.1371/journal.pone.0076057

**Published:** 2013-10-11

**Authors:** Lu Wang, Qian Jiang, Jiang Chu, Li Lin, Xiao-Guang Li, Gao-Shang Chai, Qun Wang, Jian-Zhi Wang, Qing Tian

**Affiliations:** 1 Department of Pathophysiology, Key Laboratory of Ministry of Education of Neurological Diseases, Tongji Medical College, Huazhong University of Science and Technology, Wuhan, China; 2 Department of Physiology and Neurobiology, Xinxiang Medical University, Xinxiang, China; Virginia Commonwealth University, United States of America

## Abstract

Accumulation of microtubule-associated protein tau has been observed in the brain of aging and tauopathies. Tau was observed in microglia, but its role is not illustrated. By immunofluorescence staining and the fractal dimension value assay in the present study, we observed that microglia were activated in the brains of rats and mice during aging, simultaneously, the immunoreactivities of total tau and the phosphorylated tau were significantly enhanced in the activated microglia. Furtherly by transient transfection of tau40 (human 2N/4R tau) into the cultured rat microglia, we demonstrated that expression of tau40 increased the level of Iba1, indicating activation of microglia. Moreover, expression of tau40 significantly enhanced the membranous localization of the phosphorylated tau at Ser396 in microglia possibly by a mechanism involving protein phosphatase 2A, extracellular signal-regulated kinase and glycogen synthase kinase-3β. It was also found that expression of tau40 promoted microglial migration and phagocytosis, but not proliferation. And we observed increased secretion of several cytokines, including interleukin (IL)-1β, IL-6, IL-10, tumor necrosis factor-α and nitric oxide after the expression of tau40. These data suggest a novel role of human 2N/4R tau in microglial activation.

## Introduction

The ubiquitously distributed microglia are the representative of immune cells in the relatively immune-privileged central nervous system (CNS) and account for about 10% of the total glial population in the brain [1]. They are recognized to be involved in innate immunity and surveillance of the parenchyma [2], [3]. Microglia are sensitive to brain injury and disease, altering their morphology and phenotype to adopt a so-called activated state in response to brain insults. Activated microglia phagocytose the dying cells and debris and/or release some cytokines to maintain the homeostasis of microenvironment for supporting the injured neurons [4]. Thus as an active sensor and monitor in the brain, activation of microglia is beneficial for the neuronal survival. However, lots of reports also implicated the neurotoxic roles of microglia in neurodegenerative diseases, such as Alzheimer's disease (AD) [5], [6], in which aging is the most important risk factor.

AD is characterized pathologically by extracellular senile plaques, intracellular neurofibrillary tangles (NFTs) and neuroinflammation [7], [8], [9]. Microglia are found in a highly activated state in close anatomical proximity to senile plaques in AD brains, where they secrete numerous pro-inflammatory cytokines and chemokines [9]. Thus it is thought that amyloid β (Aβ) deposits, the major component of senile plaques, constitute a chronic inflammatory stimulus triggering long-lasting activation of microglia that results in the production of neurotoxic substances, which contribute to the onset of neurodegeneration [10]. However, the cognitive impairment of AD does not correlate with Aβ load but with presence of neurofibrillar pathology evident as tau-positive structures such as neuropil threads, neurofibrillary tangles and neuritic plaques [11], [12], [13], [14].

Tau, as the major microtubule-associated protein promoting the assembly and stabilization of microtubule, reduces its ability of stabilizing microtubule and leads to the disruption of the cytoskeletal arrangement when hyperphosphorylated [15], [16]. Increased tau accumulation was reported in the brains of aging and several tauopathies including AD [17], [18], [19], [20], [21], [22]. Tau pathology was found exacerbated by lipopolysaccharide (LPS)-induced inflammation [23], [24].

In the adult human brains, alternative splicing results in the appearance of six tau isoforms, which contain, respectively, 0, 1 or 2 amino-terminal inserts and 3 or 4 microtubule-binding repeats (0N/3R, 0N/4R, 1N/3R, 1N/4R, 2N/3R and 2N/4R). Tau was first found localized in neurons, specifically to axons [25], and later studies showed its presence in the somatodendritic compartment [26]. Tau was subsequently found in glia [26], [27], and since then numerous studies have revealed abnormal accumulations of glial tau in various neurodegenerative diseases. In microglia tau assumes a particular conformation that is more readily identified by conformation-sensitive tau antibodies like Tau-66 and Tau-2 and is overlooked by tau antibodies such as Tau-5 [28], [29], [30]. Futhermore, since not all microglia stain with Tau-66, it is likely that this conformation of tau is a marker for a particular pathological state. Tau-2 shows reactive microglia and Tau-66 shows from the seemingly non-reactive to fully reactive microglia and suggests that this change in tau conformation occurs early in the microglial activation process [29]. These studies indicated the special role of tau in microglia, but no more research furtherly explains the effects of tau in microglia and its features, including the difference between microglial tau and that in neuron, astrocytes or oligodendrocytes, and the relations between the conformation and modification of microglial tau with the morphous and function of microglia.

In this study, we observed that microglia were activated in rats and mice during aging by immunofluorescence staining and the fractal dimension value assay. To explore the role of tau in microglia, we observed the immunoreactivities to total tau and the phosphorylated tau were significantly enhanced in activated microglia. As 4R tau is increased in the NFTs isolated from AD patients [31], [32], and the human tau40 (2N/4R, the longest isoform of human tau) transgenic mice recapitulate features of known neurodegenerative diseases, including AD and other tauopathies [33], we expressed tau40 in cultured rat microglial cells by transient transfection to study the role of tau in microglia during aging *in vitro* and demonstrated that expression of tau40 induced increasing of Iba1, indicating microglial activation. Furthermore, expression of tau40 significantly enhanced the membranous localization of the phosphorylated tau at Ser396 in microglia possibly by a mechanism involving protein phosphatase 2A (PP2A), extracellular signal-regulated kinase (ERK) and glycogen synthase kinase-3β (GSK-3β). It was also found expression of tau40 promoted microglial migration and phagocytosis, but not proliferation. And the secretion of several cytokines, including interleukin (IL)-1β, IL-6, IL-10, tumor necrosis factor-α (TNF-α) and nitric oxide (NO), were enhanced after tau40 expression. We have previously reported that expression of tau renders the cells more resistant to the chemically induced cell apoptosis [34], proving the new role of tau in cellular signal transduction rather than cytoskeleton. As both tau and activated microglia are increased in the brain during aging, the current data disclose the role of increased tau in microglial activation, which has not been reported previously.

## Materials and Methods

### Ethics Statement

All experimental procedures were approved by the Animal Care and Use Committee of Huazhong University of Science and Technology and were performed in compliance with National Institutes of Health guidelines for the ethical use of animals.

### Antibodies, chemicals and plasmids

All primary antibodies employed in the study are listed in Table 1. The antibodies of tau used in this study (R134d, Tau46, AT8, pS396 and pT231) recognize tau from rat, human and mouse. Anti-rabbit, anti-mouse IgG conjugated to IRDye (800 CW) were purchased from LI-COR Biosciences (Lincoln, NE, USA). Oregon Green 488-conjugated goat anti-rabbit IgG and Rhodamine Red-X-conjugated goat anti-rabbit/mouse IgG were purchased from Molecular Probes (Eugene, OR, USA). Bicinchoninic acid (BCA) protein detection kit was purchased from Pierce Chemical Company (Rockford, IL, USA). ELISA kits were purchased from R&D Systems (Minneapolis, MN, USA). Lipofectamine 2000, carboxylate-modified 1 µm yellow-green fluorescent microspheres were purchased from Invitrogen (San Diego, CA, USA). Cell culture media were purchased from Gibico (Grand Island, NY, USA). NO assay kit, membrane and cytosol protein extraction kit were purchased from Beyotime Institute of Biotechnology (Nantong, Jiangsu, China). The enhanced green fluorescent protein (EGFP) labeled human tau40 (441 amino acids) was a kind gift from Dr. Fei Liu from Jiangsu Key Laboratory of Neuroregeneration (Nantong, Jiangsu, China). The red fluorescent protein (RFP) DsRed labeled human tau40 (441 amino acids) was constructed by ourselves. All of the other chemicals were the highest purity available commercially.

**Table 1:** Primary antibodies employed in this study and their properties.

Antibody	Specificity	Type	Dilution	Source
Iba1	C-terminus of Iba1	pAb	1∶1000 WB 1∶ 200 IF	Wako (Osaka, Japan)
R134d	Total tau	pAb	1∶1000 WB	From Dr. Iqbal (New York State Institute for Basic Research in Developmental Disabilities, USA)
Tau 46	Phosphorylation independent epitope in amino acids 404–441 (human)	mAb	1∶200 IF	Sigma (St. Louis, MO, USA)
AT8	Phosphorylated tau at both Ser202/Thr205	mAb	1∶200 IF	Thermo (Rockford, Illinois, USA)
pS396	Phosphorylated tau at Ser396	pAb	1∶1000 WB 1∶200 IF	SAB (Pearland, TX, USA)
pT231	Phosphorylated tau at Thr231	pAb	1∶1000 WB	SAB (Pearland, TX, USA)
pan-Cadherin	C-terminus of pan-Cadherin	pAb	1∶1000 WB	Abcam (Cambridge, UK)
DM1A	α-tubulin	mAb	1∶1000 WB	Abcam (Cambridge, UK)
ERK	Total ERK1/ERK2	pAb	1∶1000 WB	Cell Signaling (Danvers, MA, USA)
p-ERK	Phosphorylated ERK1/ERK2 at Thr202/Tyr204	pAb	1∶1000 WB	Cell Signaling (Danvers, MA, USA)
PP2Ac	PP2A C subunit	mAb	1∶1000 WB	Millipore (Billerica, MA, USA)
p-PP2Ac	Phosphorylated PP2Ac at Tyr307	pAb	1∶1000 WB	Abcam (Cambridge, UK)
GSK-3β	Total GSK-3β	pAb	1∶1000 WB	SAB (Pearland, TX, USA)
pS9- GSK-3β	Phosphorylated GSK-3β at Ser9	pAb	1∶1000 WB	Cell Signaling (Danvers, MA, USA)

mAb, mouse monoclonal antibody; pAb, rabbit polyclonal antibody; WB, Western blotting; IF, immunofluorescence.

### Animals and treatments

SD rats (male, 4 months old and 14 months old) were obtained from the Experimental Animal Center of Tongji Medical College, Huazhong University of Science and Technology. C57BL/6 mice (male, 3 months old and 12 months old) were purchased from the Experimental Animal Center of Wuhan University. All the animals were kept under standard laboratory conditions (24±1°C, 12 h light/12 h dark cycle, light cycle begins at 06:00) with unrestricted access to food. For immunohistochemical studies, rats and mice were deeply anesthetized with intraperitoneal injection of chloral hydrate (360 mg/kg) and perfused through the aorta with 100 ml 0.9% NaCl followed by 400 ml phosphate buffer saline (PBS) containing 4% paraformaldehyde (pH 7.2, 4°C).

### Cell culture and plasmids transfection

Rat microglia were purchased from Sciencell Research Laboratory, California, USA. They were grown at 37°C in 5% CO_2_ in DMEM (high glucose) supplemented with 10% FBS, penicillin at 100 units/ml and streptomycin at 100 µg/ml. Media were changed every 2 to 3 days after plating. The adherent cells were digested by D-Hank's containing 0.25% trypsogen, plated in cell plates at suitable densities, and incubated at 37°C in 5% CO_2_ until treatment. Vector and tau40 plasmids were transfected into microglia using Lipofectamine 2000 according to the protocol of the manufacturer (Invitrogen). At 24 h after transfection, cells were collected and used for further studies. By counting of the plated cells, the transfection efficiencies of the plasmids (vector and tau40) in this study were about 40%.

### Immunofluorescence

Brains of rats or mice were removed and postfixed in perfusate overnight and then cut into sections (25 µm) by vibratome (Leica, VT1000S, Germany). The sections were collected consecutively in phosphate buffer (PB) for immunofluorescence. They were incubated overnight at 4°C with primary antibodies.

For cell studies, cells were cultured on coverslips and fixed with 4% paraformaldehyde for 20 min, permeabilized with 0.5% Triton X-100, and then incubated with primary antibodies overnight at 4°C.

After washing with PBS, brain sections or cells were subsequently incubated with Rhodamine Red-X- or Oregon Green 488-conjugated secondary antibodies (1∶1000) for 1 h at 37°C. The images were observed by laser confocal microscope (LSM710, Zeiss, Germany) and the fluorescence intensity was analyzed by the software affiliated. The fractal dimension value of microglia was analyzed by Image Pro Plus 6.0 (Media Cybernetics, Silver Spring, MD, USA).

### Western blotting

We harvested the cells and lysed them with sample buffer containing 50 mM Tris-HCl (pH 7.6), 2% sodium dodecyl sulfate (SDS), 10% glycerol, 3 mM PMSF and 0.2% bromophenol blue. After they had been boiled for 10 min, the cell lysates were ultrasonic processed for 15 s. The protein concentration of the cell lysates was estimated by BCA kit. The proteins were separated by 10% SDS-polyacrylamide gel electrophoresis and transferred to nitrocellulose (NC) membrane, blocked in TBS (50 mM Tris-HCl, pH 7.6, 150 mM NaCl) containing 5% skimmed milk for 40 min. After they had been washed with distilled water, the blots were incubated with primary antibodies at 4°C overnight. After they had been washed 3 times with TBS containing Tween-20 (0.2%), the blots were probed by using IRDye 800CW-conjugated secondary antibodies (1∶10000) for 1 h. The blots were visualized using infrared fluorescence imaging and the intensity of them was quantified by Odyssey infrared imaging system (Li-Cor Bioscience, Lincoln, NE, USA). The levels of the proteins were expressed as relative levels of the sum optical density against controls.

### Membranous and cytosolic protein extraction

Rat microglia were resuspended in solution A contained 1 mM PMSF and mechanically dissociated by trituration. They were centrifuged at 700 g for 10 min at 4°C to remove unbroken cells and nucleis. Supernatant were centrifuged at 14000 g for 30 min at 4°C to precipitate membrane fragments. Supernatant were collected as cytosolic protein, sediment were centrifuged at 14000 g for 10 s at 4°C and added 1/5 (v/v) of solution B with frequent vortexing for 5 s and incubated on ice for 10 min, then vortexing and ice-incubating were repeated. The solution was centrifuged at 14000 g for 5 min at 4°C and supernatant were collected as membrane protein.

### *In vitro* scratch assay

Rat microglia were cultured as confluent monolayers in culture dishes and transfected with vector/tau40-encoding EGFP fusion protein for 24 h, and then scratched with a 100 µl pipette tip. The scratched monolayers were washed twice to remove non-adherent cells and media were changed with DMEM [35], [36]. Then the cells were observed by laser confocal microscope (LSM510, Zeiss, Germany) and analyzed of the cell migration at 0 h, 6 h, 12 h, and 24 h separately. Phase contrast and fluorescence images were taken at the different time points until the wound closed. The wounded area was defined in each image by positioning lines in correspondence to the original scratch and the following data were analyzed by Image Pro Plus 6.0.

### Phagocytosis assay

Rat microglia were collected and plated in cell plates and then transfected with vector/tau40-encoding RFP fusion protein. At 24 h after transfection, media were changed with DMEM. The cells were then treated at 37°C for 30 min with 1 µm yellow-green fluorescent microspheres. After incubation, microspheres were washed with PBS and then fixed with 4% paraformaldehyde for 20 min. The fluorescent microspheres were excited very efficiently using the 488 nm spectral line of the argon-ion laser and had exceptionally intense fluorescence. Analysis of phagocytized particles had been carried out by fluorescence microscope (Zeiss, Germany) and quantitative flow cytometer (BD Biosciences, USA).

### ELISA and NO assay

For ELISA assay, supernatant of rat microglial cells were collected as sample. 50 µl of diluent 1× and 50 µl of standard or sample were added to each well. After being incubated at 37°C for 30 min, each well were aspirated and washed for 30 s ×5 with wash solution (400 µl). After the last wash, any remaining wash buffer was removed by aspirating or decanting. Then 100 µl of rat IL-1β/IL-6/TNF-α/IL-10 conjugate was added to each well. After being incubated at 37°C for 30 min, aspiration and washing were repeated. 100 µl of substrate solution was added to each well. After being incubated at 37°C for 10 min (protect from light), 100 µl of stop solution was added to each well. The color in the wells should change from blue to yellow. We used a microplate reader (Biotek, Winooski, VT, USA) set to 450 nm to determine the optical density of each well within 30 min. A standard curve was prepared from standard dilutions of cytokines in duplicate. The cytokine concentration in each sample was determined from the standard curve.

The level of NO was measured according to the protocol of the manufacturer (Beyotime). We used a microplate reader set to 540 nm to determine the optical density of each well within 30 min. A standard curve was prepared from standard dilutions of NO in duplicate. The concentration of NO in each sample was determined from the standard curve.

### Dot blot

At 24 h after transfection, the medium of rat microglial cells was collected and centrifuged at 300 g for 5 min. 5 µl of supernatant was applied to the marked circle on NC membrane and dried before being blocked. Then the NC membranes were incubated with antibodies as introduced in Western blotting.

### Statistical analysis

Data were analyzed by using SPSS 12.0 statistical software (SPSS, Chicago, IL, USA). All data were expressed as means ± S.D.. Statistical significance was determined by Student's two-tailed t-test with 95% confidence.

## Results

### Microglia are activated during aging and tau accumulates in activated microglia

**Figure 1:**
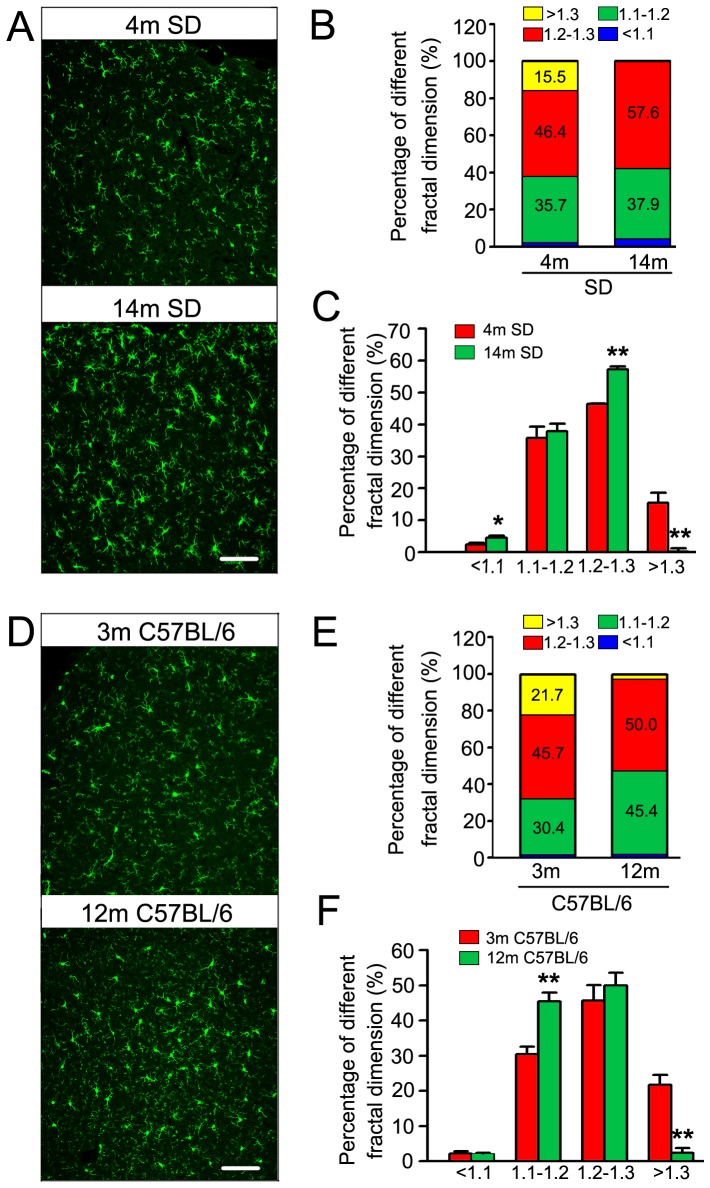
Activation of microglia in the brains of rats and mice with aging. Microglia in the cortex of 4- and 14-month-old SD rats (A), 3- and 12-month-old C57BL/6 mice (D) were immunostained by Iba1, a marker of microglia (Scale bar = 100 µm). The fractal dimension value analysis was used to evaluate the activation of microglia in the brains of different group of animals. Lower fractal dimension value indicates higher activity of microglia. We divided the fractal dimension value of the microglia into four grades (>1.3, 1.2–1.3, 1.1–1.2, <1.1), and the percentages of microglia with different grades were shown in B and E, the differences of the same grade between the different age of animals were shown in C and F (n>43 cells/group). Data were presented as means ± S.D.. * *p*<0.05, ** *p*<0.01 versus 4-month-old SD rats/3-month-old C57BL/6 mice.

Microglial activation has been observed in the brains of aging [37], [38] and AD patients [39]. By using the antibody of Iba1, a specific marker of microglia [40], we stained the brain slices of rats and mice at different ages (Figure 1A, D). Normally, ramified microglia with small soma and arborescent processes are resting microglia. Ameboid microglia with larger soma and less processes are regarded as activated microglia [41], [42], [43]. By fractal dimension value analysis for evaluation of microglial activation [44], [45], we found more microglia with lower fractal dimension value in elder rats and mice (Figure 1B, C, E, F), indicating microglial activation in the brains of the elder animals.

**Figure 2:**
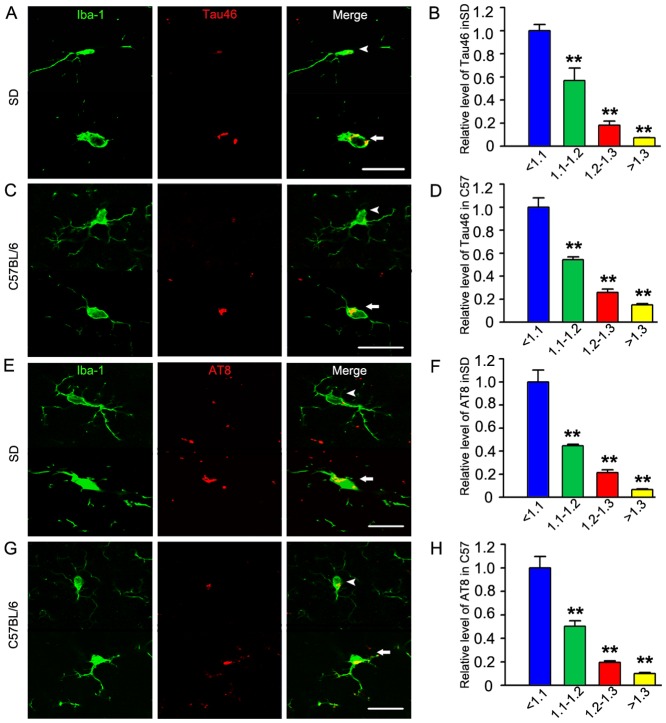
Both total tau and phosphorylated tau increase in activated microglia. Microglia in the brain slices were co-immunostained with Iba1 (green) and Tau46 (an antibody recognizing total tau) or AT8 (an antibody recognizing phosphorylated tau at Ser202/Thr205) (red). In contrast with the ramified microglia (arrow heads), ameboid microglia (arrows) showed increased total tau (A, C) and the phosphorylated tau (E, G) (Scale bar = 20 µm). The relative levels of integrated optical density (IOD) of Tau46/AT8 in microglia with different grades of fractal dimension value were shown in B, D, F, H. Data were presented as means ± S.D.. ** *p*<0.01 versus microglia with fractal dimension value <1.1.

Previous studies demonstrated that level of tau protein was increased in the aged brain and the increase was more significant in the brain of AD [46], [47], [48], [49]. To measure the level of tau in microglia, we double-stained the brain slices with the antibody recognizing total tau (Tau46) or phosphorylated tau (AT8) and the antibody recognizing microglia (Iba1). Positive immunoreactions to Tau46 and AT8 were observed in different types of microglia (Figure 2A, C, E, G). From Figure 1B, E and Figure 2B, D, F, H, we found the level of total tau (recognized by Tau46) in microglia of the 14 months old rats is 1.18 times of that in 4 months old rats, and the phosphorylated tau (recognized by AT8) is 1.15 times. The level of total tau of microglia in 12 months old C57BL/6 mice is 1.19 times of that in 3 months old mice, and the phosphorylated tau is 1.21 times. However, the levels of total tau and phosphorylated tau in microglia with lowest fractal dimension value were highest (Figure 2B, D, F, H).

### Expression of tau40 activates microglia with membranous accumulation of phosphorylated tau

**Figure 3:**
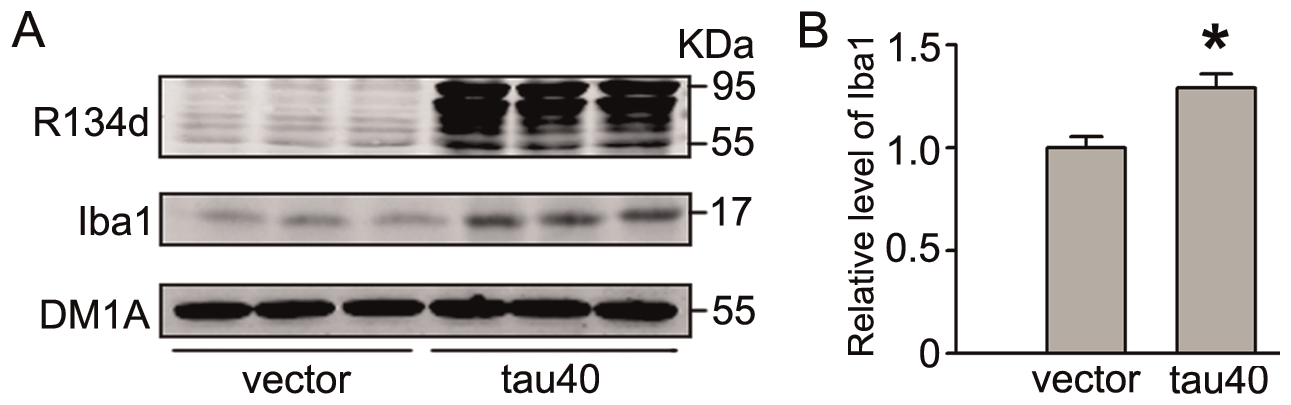
Expression of tau40 induces increased Iba1. In cultured rat microglia, the plasmid of human tau40 (441 amino acids) or vector was transfected for 24 h. Then the levels of total tau probed by R134d and Iba1, a marker of microglial activation, were measured by Western blotting (A) and quantitative analysis (B) respectively. The alteration of Iba1 was normalized against DM1A. The experiments were repeated at least three times and data were presented as means ± S.D.. * *p*<0.05 versus vector-transfected cells.

**Figure 4:**
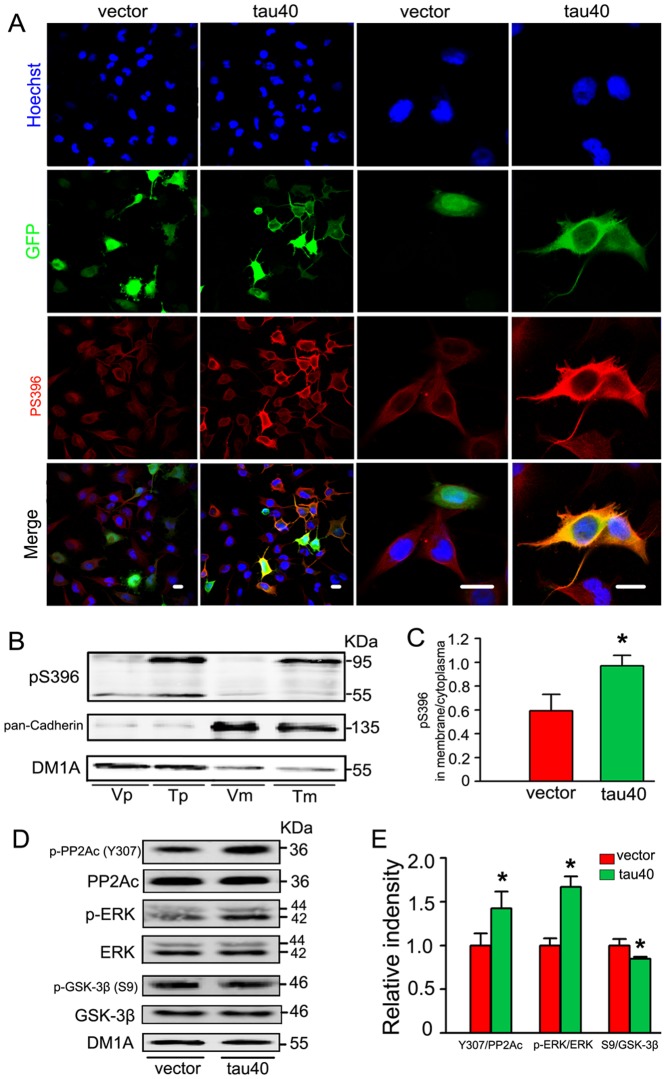
Expression of tau40 induces membranous accumulation of phosphorylated tau, simultaneously with inhibition of PP2A and activation of ERK and GSK-3β. In cultured rat microglial cells, the plasmid of human tau40-EGFP (T) or vector-EGFP (V) was transfected. 24 h later, triple immunofluorescence imaging was performed. The nucleus was stained with Hoechst (blue) and the phosphorylated tau was probed by pS396 (an antibody recognizing phosphorylated tau at Ser396) (red). Then cells were observed by confocal microscope (A) (Scale bar = 20 µm). The membranous (m) and cytoplasma (p) fractions were isolated as described in the method and the level of pS396 in the two fractions was analyzed by Western blotting (B) and quantitative analysis (C). The levels of PP2Ac, p-PP2Ac (Y307), ERK, p-ERK, GSK-3β and p-GSK-3β (S9) were probed and measured by Western blotting (D) and quantitative analysis (E). The data were representative of three independent experiments and were presented as means ± S.D.. * *p*<0.05 versus vector-transfected cells.

To verify the effects of tau on microglial activation, we measured the level of Iba1 after transient expression of tau40 or the vector (as control) in the cultured rat microglia. The level of Iba1 was significantly increased at 24 h after transfection (Figure 3), indicating the activation of microglia. By immunofluorescence staining, we noticed that the endogenous tau phosphorylated at Ser396 was uniformly distributed in the cytoplasma of the vector-transfected and un-transfected microglia, while expression of exogenous tau resulted in membranous accumulation of phosphorylated tau at Ser396 (Figure 4A). To further verify the distribution of tau in microglia, we isolated membranous fraction from cytosolic fraction after transfection of tau40 or vector and measured the level of tau in different fractions. DM1A and pan-Cadherin were used as makers of cytoplasma and membrane, respectively. Expression of tau40 significantly increased the phosphorylation of tau at Ser396 in membrane fraction (Figure 4B, C). To investigate the possible mechanism, we measured the activity-related phosphorylation levels of PP2A, ERK and GSK-3β, which are highly involved enzymes in tau phosphorylation (Figure 4D). The levels of phosphorylated PP2A catalytic subunit (PP2Ac) at Thr307 and phosphorylated ERK at Thr202/Tyr204 were significantly increased, while the phosphorylated GSK-3β at Ser9 was decreased, indicating inhibition of PP2A and activation of ERK and GSK-3β (Figure 4E). These data together suggested that tau40 activated microglia and caused membranous accumulation of phosphorylated tau possibly by a mechanism involving PP2A, ERK and GSK-3β.

### Expression of tau40 promotes migration, phagocytosis and secretion of microglia

**Figure 5:**
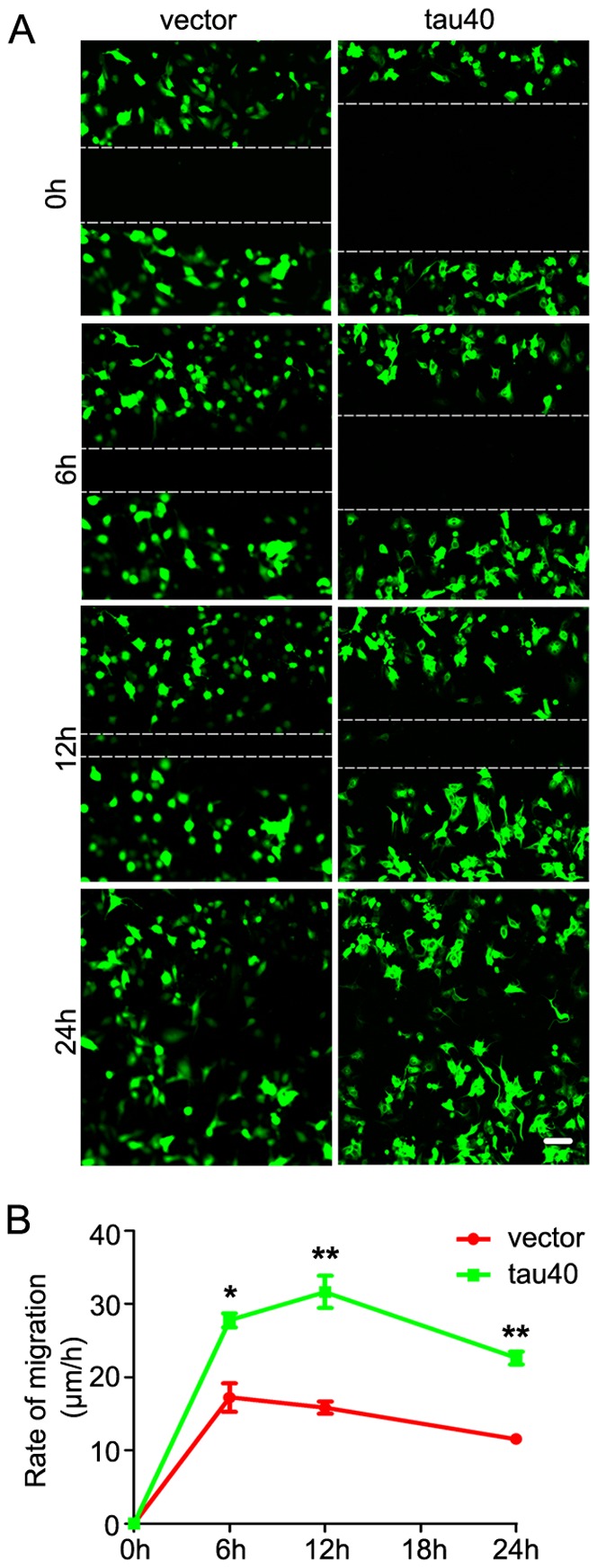
Expression of tau40 promotes migration of microglia. In cultured rat microglia, the plasmid of human tau40-EGFP or vector-EGFP was transfected for 24 h. Then *in vitro* scratch assay was performed and the images of migration were captured at 0 h, 6 h, 12 h and 24 h after scratching with confocal microscope (A) (Scale bar = 100 µm). The migration rate of microglia was quantified by the distance that the EGFP positive cells moved from the edge of the scratch towards the center per hour. The average migration rates of the EGFP positive microglia in 0–6 h, 6–12 h and 12–24 h were calculated from three independent experiments (B). Data were presented as means ± S.D.. * *p*<0.05, ** *p*<0.01 versus vector-transfected cells.

Actived microglial cell presents high ability of migration, phagocytosis and secretion [3]. To explore the role of tau40 in microglial migration, we used the *in vitro* scratch assay as reported and calculated the average migration rate in different time intervals and the total migration distance within 24 h [50]. We observed that expression of tau40 accelerated microglial migration toward the center of the wound and the average speeds were ∼1.6-fold in 0–6 h, ∼2.0-fold in 6–12 h and 12–24 h of the control (vector transfected) microglia. In 24 h, the control microglia moved about 277 µm, while the microglia with expression of tau40 moved about 543 µm (Figure 5). By cell counting, no difference in proliferation was seen between two groups (not shown). These data indicated that tau40 accelerated microglial migration in culture.

**Figure 6:**
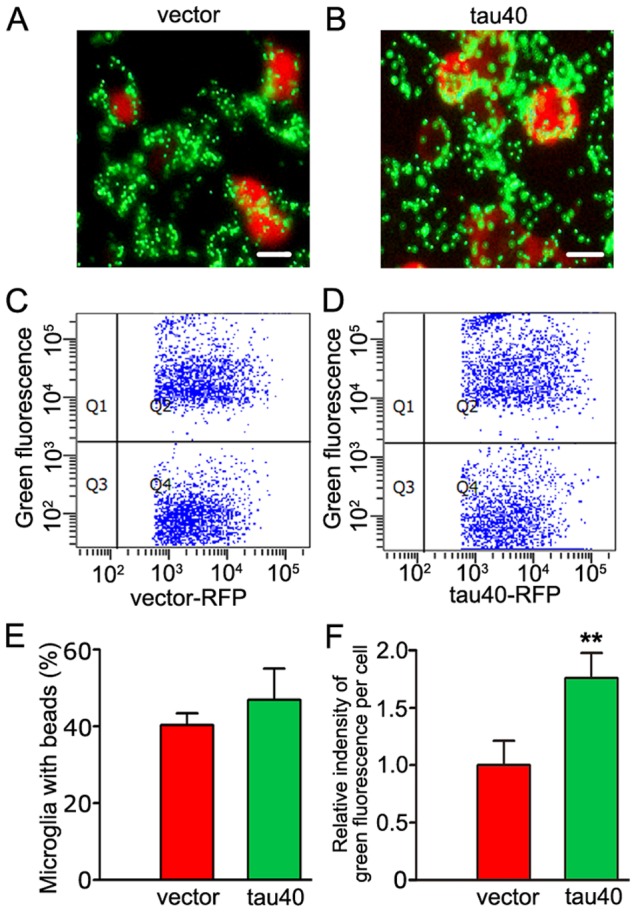
Expression of tau40 enhances phagocytosis of microglia. In cultured rat microglia, the plasmid of human tau40-RFP or vector-RFP was transfected. 24 h later, yellow-green fluorescent beads with the diameter of 1 µm were added into the medium. After being incubated at 37°C for 30 min, the phagocytosis of microglia was observed by fluorescence microscopy (A, B) (Scale bar = 20 µm) and quantitatively analyzed by flow cytometry (C, D) respectively. The percentage of transfected microglia containing green fluorescence (E) and the average green fluorescent intensity of single transfected microglia (F) were calculated from three independent experiments. Data were presented as means ± S.D.. ** *p*<0.01 versus vector-transfected cells.

Microglial cells are recognized phagocytes in CNS and this function is important for the normal brain during the development, pathology and regeneration of brain [51], [52]. To explore the effect of tau40 on phagocytosis of microglia, we constructed the plasmid of vector/tau40-encoding RFP fusion protein and transfected the plasmids into the cultured rat microglial cells for 24 h, and then 1 µM yellow-green fluorescent microspheres were added and incubated at 37°C for 30 min. It was observed that microglia expressing tau40 phagocytized more microspheres than vector-transfected and un-transfected cells by fluorescence microscope, and there was no difference between vector-transfected and un-transfected cells (Figure 6A, B). We furtherly used quantitative flow cytometry to analyze the percentage and the index of phagocytosis (Figure 6C, D). The results showed that single tau40-expressing cell phagocytized more fluorescent beads than the vector-transfected cell. However, there was no significant difference in the percentage of the cells phagocytized beads between two groups (Figure 6E, F). These data suggested that expression of tau40 enhanced the phagocytic capacity of microglia.

**Figure 7:**
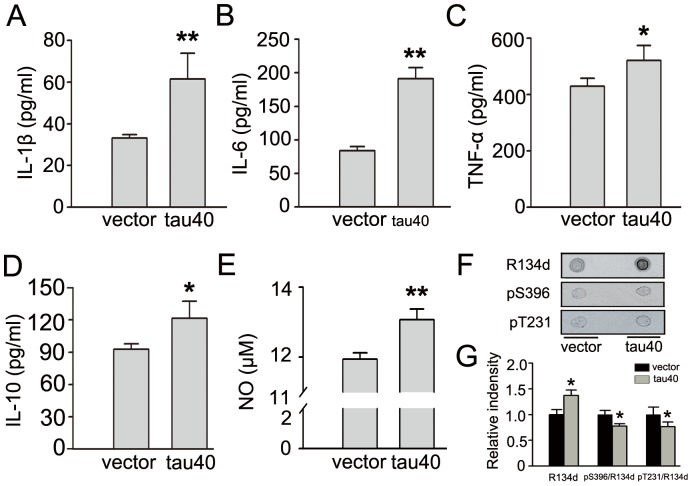
Expression of tau40 increases the levels of inflammatory cytokines and tau in the medium. In cultured rat microglial cells, the plasmid of human tau40-EGFP or vector-EGFP was transfected. 24 h after transfection, levels of IL-1β (A), IL-6 (B), TNF-α (C) and IL-10 (D) in the supernatant of medium were assessed by ELISA. And the concentration of NO (E) was represented by nitrite which was determined by Griess reaction. The release of tau (R134d) and its phosphorylation at Ser396 (pS396) and Thr231 (pT231) in the medium were assessed by dot blot (F) and quantitative analysis (G). The experiments were repeated at least three times, and data were presented as means ± S.D.. * *p*<0.05, ** *p*<0.01 versus vector-transfected cells.

Actived microglia could release cytokines, which have been classified based on their actions in peripheral tissues as either anti-inflammatory cytokines, such as IL-4, IL-10, or pro-inflammatory cytokines, such as IL-1β, IL-6, TNF-α and NO [53]. We measured the levels of inflammatory cytokines in the medium at 24 h after transfection of tau40 or the vector. Obvious increased levels of IL-1β, IL-6, TNF-α, IL-10 and NO were detected in the medium after tau40 transfection (Figure 7A–E). Recent studies suggested that tau could be released from viable cells [54], [55], [56]. To verify if tau could be released from microglia, we measured the levels of total tau (recognized by R134d) and phosphorylated tau (recognized by pS396 and pT231) in the medium by dot blot (Figure 7F). It was shown that both vector and tau40 transfected cells released tau, and the later released more tau. By calculating the ratio of phosphorylated tau, the levels of released tau phosphorylated at Ser396 and Thr231 epitopes were lower in tau40 expressing cells (Figure 7G). Taken together with previous data (Figure 4A, B, C), it is possible that tau40 transfected cells released unphosphorylated tau and kept more phosphorylated tau in themselves.

## Discussion

In present study, we found that microglia were activated in the brains of rats and mice with aging, simultaneously the increase of tau was shown in the activated microglia. Further studies in the cultured rat microglia demonstrated that expression of tau40 induced activation of microglia with accelerated migration, enhanced phagocytosis, increased secretion of inflammatory cytokines and unphosphorylated tau, and the activation of ERK and GSK-3β. Our data suggest that tau40 may serve as an upstream factor to trigger microglial activation during aging and AD.

Tau is often detected in the axon and dendrite of neuron [57], [58]. It has recently been identified on the plasma membrane [59], [60], [61]. Here, our data showed that tau40 in microglia induced increased phosphorylation of tau at Ser396, which is predominantly located on the plasma membrane.

Some kinases in microglia could phosphorylate tau proteins, such as ERK1/2 and GSK-3β [62]. ERK is a mitogen-activated protein kinase (MAPK) and and phosphorylation of ERK is an early event in the activation of microglia [63], [64]. GSK-3 is an important serine/threonine protein kinase phosphorylating tau at the majority of AD-related sites [65]. The role of GSK-3β in inflammation was reported firstly in 2005 [66]. Activation of GSK-3β modulates the nuclear translocation of nuclear factor-κB (NF-κB) and cAMP-response element binding protein (CREB) by enabing CREB binding protein (CBP) to bind both transcriptional factors, which facilitates nuclear translocation and increases the production and transcription of pro-inflammatory cytokines [67]. PP2A is the main protein phosphatase involved in tau phosphorylation, and the activity of PP2A is decreasesd in AD [68], [69], [70]. In present study, the activation of ERK and GSK-3β and inhibition of PP2A were detected in tau40 transfected microglial cells, which might contribute to the increased phosphorylation of tau at Ser 396. The mechanism involved in activation of ERK and GSK-3β and inhibition of PP2A in microglia requests further studies.

After activation, microglial cell will migrate to the site of injury or inflammation [71]. Cell motility is a dynamic process driven by structurally and functionally coordinated reorganization of the actin cytoskeleton [72], [73]. Many of the pro-migratory factors are members of the chemokine family. Therefore, we tested whether tau40 could regulate the migration of the cultured microglia and found that tau40 remarkably accelerated the migration. It is reported that tau may act to facilitate neuronal migration synergistically [74]. Phagocytosis is another important function of microglia. In the AD brain, microglia increased and clustered in and around Aβ deposits [75], [76]. In a P301S tauopathy mouse model, exhibiting certain aspects of neurofibrillary tangle formation of AD, early microglial activation was associated with loss of synapses [77]. There were also evidences indicating that accumulation of microglia in AD might be protective and promote Aβ clearance [39], [78], [79]. In our current study, we found tau40 transfected microglia showed stronger phagocytosis. It is currently not understand how tau40 enhances phagocytosis of microglial cells.

Gene array studies have shown that various cytokines are elevated in aged rodent brains [80], [81], and the protein levels of both anti-inflammatory cytokines (e. g. IL-10) and pro-inflammatory cytokines (e.g. IL-1β, TNF-α and IL-6) are elevated during aging [82]. In the present study tau40 increased the levels of both anti-inflammatory cytokines (IL-10) and pro-inflammatory cytokines (IL-1β, TNF-α, IL-6 and NO). IL-1β has long been recognized as a key mediator of immune and inflammatory responses during infection. In the CNS, IL-1β is mainly expressed and released by microglia, although astrocytes and neurons may also contribute to production of IL-1β, particularly in the late phase after excitotoxicity [83]. In the early phase of CNS injury, microglia release IL-6 [84], a multifunctional cytokine with diverse actions including the regulation of inflammation, immune responses and cell differentiation. TNF-α could be produced by neurons, astrocytes, and microglia in the CNS [85], and low level of TNF-α has neuroprotective consequences [86]. NO is generated by inducible nitric oxide synthase (iNOS) in microglia, and a wide variety of neurologic injuries or diseases are associated with the induction of iNOS and the generation of NO by microglia [87], [88]. Compared with LPS treatment, a classical activator of microglia, the magnitudes of changes in inflammatory factors induced by tau40 in this study were relatively modest. We have reported that overexpression of tau can protect the cells from an acute apoptosis [34]. It will be interesting in further studies to investigate whether and how the tau-induced cytokines release from microglia affect the viability of neurons or other types of glial cells or microglia itself.

In AD the mechanism involved in the spreading of tau pathology is unknown. It is speculated that tau, the primary component of NFTs, is released following neuronal death, allowing it to be taken up by neighbouring cells including microglia [89]. Tau released from healthy neurons could be a physiological process that might be disrupted in diseased brain. Total tau and hyperphosphorylated tau in cerebrospinal fluid (CSF) have been shown to correlate with neurofibrillary pathology in AD [90], [91]. Thus it is possible that microglia can phagocytize tau released by neuron and/or glia, which leading to the activation of microglia during aging.

It was reported that tau assumes a particular conformation that is more readily identified by conformation-sentitive tau antibodies like Tau-66 and Tau-2 and is overlooked by tau antibodies such as Tau-5 in microglia [28], [29], [30]. Tau-2 shows reactive microglia and Tau-66 shows from the seemingly non-reactive to fully reactive microglia, which suggests that this change in tau conformation occurs early in the microglial activation process [29]. As Tau-2 positive microglia-like cells (TPMCs) do not react with conventional anti-tau antibodies and is devoid of fibrils composed of hyperphosphorylated tau, TPMCs are also thought to represent a conformational state that can be reversibly diminished upon exposure to a detergent and may instead represent a secondary event shared with ischemic/inflammatory foci. Thus, it is highly debatable whether tau proteins are the principal constituents of these TMPCs. Here, we found more total tau and phosphorylated tau in activated microglia with lower fractal dimension value. As we did not have human brain sample or human microglia cell line, we expressed tau40 in cultured rat microglial cells by transient transfection and observed increasing of Iba1 level and increased microglial migration, phagocytosis and secretion, indicating the activation of microglia after tau40 transfection. If human microglia cell cultures were used, the results may be more persuasive. Totally, all these data of this research indicate tau is important for microglial activation in aging and AD, which have not been substantially replicated in the previous literature. But the role of phosphorylated tau in microglial activation needs further investigation.
